# Giant retroperitoneal liposarcoma measuring 27 × 29 × 36 cm: a case report

**DOI:** 10.1093/jscr/rjac608

**Published:** 2023-01-10

**Authors:** Catalina Gutu, Valentin Butnari, Victor Schiopu

**Affiliations:** Department of Oncology, Nicolae Testemitanu State University of Medicine and Pharmacy, Chișinău, Moldova; General Surgery, Barking, Havering and Redbridge University NHS Trust, London, UK; Department of Oncology, Nicolae Testemitanu State University of Medicine and Pharmacy, Chișinău, Moldova

## Abstract

Retroperitoneal tumors are rare neoplasms that can reach great dimensions due to a slow growth pattern. Although these tumors rarely metastasize, they have a great risk of recurrence, and majority of times these lesions are a challenge for the surgeon. We report the case of a 63-year-old woman who presented with symptoms of large bowel obstruction and was diagnosed with a large heterogenous mass located in the retroperitoneal space. The fine needle biopsy revealed the histology of liposarcoma. The purpose of this article is to report our approach in management of this kind of tumor. Tumor size (27.1 × 29.1 × 36.1 cm) and involvement to the adjacent organs was a challenge for us in order to reach safe oncological margins. In these cases, the risk of recurrence is high; therefore, the patient should be screened at 6, 12 and 24 months post procedure. Chemotherapy or radiotherapy for this tumor is not yet defined, and if operable, surgery is the treatment of choice.

## INTRODUCTION

Retroperitoneal sarcomas (RPSs) are rare tumors, accounting for 0.1–0.2% of all malignant tumors [1]. The epidemiology of RPSs is not well described [2], although it is expected to be approximately 0.5–1 new cases per 100 000 persons per year, representing ~15% of all soft tissue sarcomas [3]. These tumors are commonly asymptomatic until they reach great dimensions. The mean size at the time of diagnosis is 15–18 cm [4]. Surgery is the mainstay and only true curative treatment for RPSs. Due to malnutrition at presentation, patients may require admission to hospital for general medical optimization, which may include parenteral nutrition, pre-habilitation and consultation with anesthesia [5]. The goal of RPS surgery is complete resection, as this is the only chance for a cure. Ideally, negative microscopic margins (R0) should be obtained. However, given the massive size of the tumor, accurate pathologic assessment of all margins on the resected tumor specimen is logistically difficult to achieve. Therefore, complete resection is often defined as macroscopically negative margins with either negative (R0) or positive (R1) microscopic margins [6]. Chemotherapy showed no benefit in relapse-free survival or overall survival [7, 8]. At present, the role of radiotherapy remains unclear. Although postoperative radiotherapy has been associated with lower local relapse rates in some retrospective series, this approach comes at the cost of substantial toxicity. If radiotherapy is considered, most experts prefer the preoperative setting as the optimal sequencing with resection [9].

## CASE REPORT

**Figure 1:**
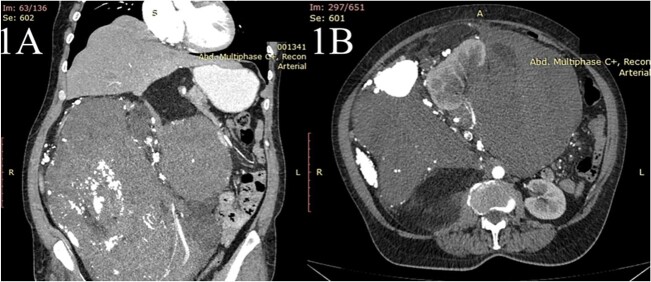
The contrast-enhanced computed tomography (CT) scan shows (**A**) a huge mass extending from the sub-hepatic region to the pelvis with the cranial displacement of the liver and (**B**) the same mass causing encasement the right kidney.

A 63-year-old woman presented with a 6-month history of progressive weight loss, nausea, vomiting, diffuse abdominal pain and constipation. An ultrasonography exam revealed a huge retroperitoneal mass. The computed tomography with intravenous contrast revealed a large heterogenous mass having multiple linear septation and HU value of −90 of 27 × 29 × 36 cm (Fig. 1A and B) originating from the retroperitoneal space, extending from the sub-hepatic space to the pelvis with a cranial displacement of the liver and a left displacement of the pancreas, stomach and the small bowel and encasement of the right kidney. Fine needle biopsy revealed liposarcoma. The preoperative assessment revealed only mild anemia (Hb = 9.4 mg/dl, erythrocytes—4.7 * 10^12^/l, hematocrit = 31%).

**Figure 2:**
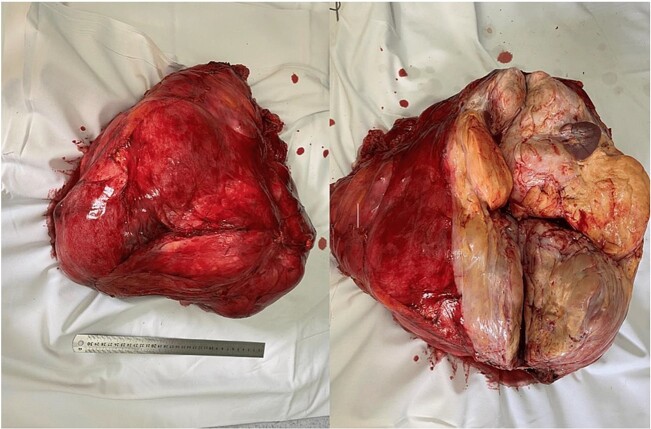
Removed specimen.

The mass was approached by a midline incision. An extension of the mass was seen on the right side of the abdomen cavity. The diaphragm, pancreas and hepatoduodenal ligament were invaded by the tumor. We aimed to resect it with preservation of the abdominal organs (Fig. 2). Unfortunately, the diaphragm was injured during the tumor resection, which resulted in a pneumothorax.

**Figure 3:**
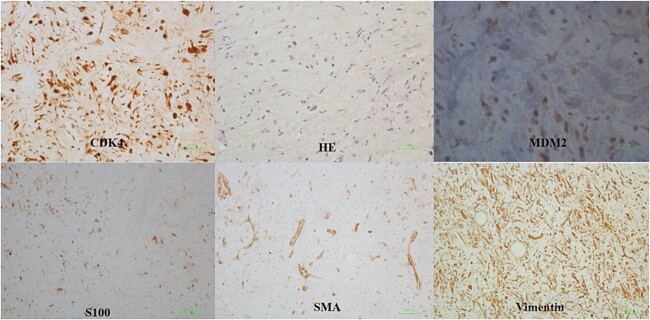
Histologic examination.

Histologic sections revealed a well-differentiated liposarcoma (Fig. 3), a sclerosing subtype. Immunohistochemistry showed a positive reaction to Cyclin dependent kinase-4 (CDK4), Mouse double minute 2 homolog (MDM2), Vimentin, S100 and smooth muscle actin (SMA).

The postoperative course was uneventful, but due to intraoperative complications—pneumothorax and major blood loss of 3600 ml, the patient’s discharge was delayed, namely, on the 13th postoperative day.

## DISCUSSION

Overall, local/abdominal recurrence is common (≈50% at 5 years overall) following resection of primary RPS. A high proportion of recurrences occur late (after 5 years), mandating ongoing follow-up [10]. Tumor characteristics, such as histological subtype, dedifferentiation grade and multifocality, have proven to be important indicators of disease-free survival. R1 margins have been associated with decreased disease-free survival, which underlies the crucial role of an aggressive surgical approach to RPS due to the importance of obtaining R0 margins [11].

Several studies suggest that tumor size does not have an impact over overall survival [4, 12], while prognostic factors for recurrent RPS have been reported as a high tumor grade, margin status and a young age [3, 13–16].

Well-differentiated liposarcoma is a locally aggressive adipocytic neoplasm with no potential for metastasis. We all know that lipomatous tumors are immunoreactive to S100; however, the adipocytic nature of the tumor is usually obvious and does not warrant an S100 stain. The morphologic distinction between benign lipoma and atypical lipomatous tumor relies on the identification of the hallmark diagnostic cells, which are the atypical hyperchromatic stromal cells and lipoblasts. The histologic types include adipocytic, sclerosing, spindle cell and inflammatory variants. When in doubt of this diagnosis, nuclear immunoreactivity of MDM2 and cyclin-dependent kinase 4 (CDK4) is confirmatory, which corresponds to amplification of these genes [17].

Well-differentiated liposarcoma is a genetically distinct group of lesions. With the exception of the spindle cell variant, all well-differentiated liposarcoma subtypes share the same genetic aberration, represented by the presence of distinctive ring and/or giant marker chromosomes. Ring chromosomes contain amplified sequences derived from the 12q13–15 chromosome region, where several proto-oncogenes including MDM2, CDK4 and HMGA2 are located. Therefore, amplification of HMGA2, MDM2 and CDK4 (as well as overexpression of the corresponding proteins) can be detected by molecular or immunohistochemical techniques in well-differentiated liposarcoma, which is a helpful confirmatory diagnostic tool. S-100 immunopositivity is helpful to highlight the presence of multivacuolated lipoblasts. [18]

Sclerosing well-differentiated liposarcoma is best recognized by the presence of scattered bizarre, hyperchromatic stromal cells set in a fibrillary collagenous background [18].

While tumor-related factors are paramount, the only intervenable predictive factor is the extent and quality of surgery [19]. The results of various studies [20–22] have been embraced by many groups as justification to routinely employ extended compartmental resections for the bulk of primary RPSs. Standardization of technical principles of oncologic resection on the basis of those employed for extremity soft tissue sarcoma has been published as consensus by a subset of European and American sarcoma specialists who advocate that ‘where barriers exist, proceed beyond the safe tissues leaving the tumor covered by the barriers; where anatomic barriers do not exist, try to use adjacent organs as a new barrier if their sacrifice is acceptable in terms of short and long-term morbidity’. Extended resection is generally limited to visceral resection of colon, kidney, distal pancreas, diaphragm, spleen and psoas. However, many large retroperitoneal tumors lie immediately adjacent to bone, duodenum and major vessels, generating bias in the definition of ‘radical’ resection. The quality of resection will be limited by the closest margin, thus, in tumors which will have other close margins along vessels and other critical structures, extended resection may not be justifiable unless indicated for complete gross resection. Therefore, careful consideration is given to clearing all ipsilateral retroperitoneal fat, treating these areas as part of the retroperitoneal ‘compartment’ and minimizing the risk of leaving behind unrecognized low-grade components of the tumor [23]. However, organs are rarely invaded at the time of primary tumor resection. Reports examining the involvement of adjacent kidney by pathologic review show that only 9% show parenchymal infiltration [24].

The aim of the extended surgery approach is to maximize the possibility of achieving a complete resection through a standardized approach based on histologic behavior and site of origin. In liposarcoma, the extended approach often includes the resection of adjacent visceral organs (mainly kidney, colon, and psoas muscle) due to the difficulties in understanding tumor boundaries, multifocality and the importance of local control. Of note, the extended approach may preserve some of the adjacent organs thanks to its different patterns of growth, with a clear separation of tumor surface from the rest of the adjacent organs. Preserving noninvaded organs does not compromise the quality of the surgical resection [19].

## Data Availability

Anonymized data can be provided for data transparency.
